# Effects of Magnesium Supplementation on Unipolar Depression: A Placebo-Controlled Study and Review of the Importance of Dosing and Magnesium Status in the Therapeutic Response

**DOI:** 10.3390/nu10081014

**Published:** 2018-08-03

**Authors:** Beata Ryszewska-Pokraśniewicz, Anna Mach, Michał Skalski, Piotr Januszko, Zbigniew M. Wawrzyniak, Ewa Poleszak, Gabriel Nowak, Andrzej Pilc, Maria Radziwoń-Zaleska

**Affiliations:** 1 Nowowiejski Hospital, 00-685 Warsaw, Poland; beataryszewska@wp.pl; 2 Department of Psychiatry, Medical University of Warsaw, 00-685 Warsaw, Poland; michal.skalski@wum.edu.pl (M.S.); piotr.januszko@wp.pl (P.J.); mariar@wum.edu.pl (M.R.-Z.); 3 Faculty of Electronics and Information Technology, Warsaw University of Technology, 00-685 Warsaw, Poland; z.wawrzyniak@ise.pw.edu.pl; 4 Faculty of Pharmacy, Medical University of Lublin, 20-093 Lublin, Poland; ewa.poleszak@umlub.pl; 5 Institute of Pharmacology, Polish Academy of Sciences, 31-343 Kraków, Poland; nowak@if-pan.krakow.pl (G.N.); nfpilc@cyf-kr.edu.pl (A.P.)

**Keywords:** unipolar depression, magnesium, pharmaco-electroencephalography, efficacy, remission

## Abstract

Animal studies using tests and models have demonstrated that magnesium exerts an antidepressant effect. The literature contains few studies in humans involving attempts to augment antidepressant therapy with magnesium ions. The purpose of our study was to assess the efficacy and safety of antidepressant treatment, in combination with magnesium ions. A total of 37 participants with recurrent depressive disorder who developed a depressive episode were included in this study. As part of this double-blind study, treatment with the antidepressant fluoxetine was accompanied with either magnesium ions (120 mg/day as magnesium aspartate) or placebo. During an 8-week treatment period, each patient was monitored for any clinical abnormalities. Moreover, serum fluoxetine and magnesium levels were measured, and pharmaco-electroencephalography was performed. The fluoxetine + magnesium and fluoxetine + placebo groups showed no significant differences in either Hamilton Depression Rating Scale (HDRS) scores or serum magnesium levels at any stage of treatment. Multivariate statistical analysis of the whole investigated group showed that the following parameters increased the odds of effective treatment: lower baseline HDRS scores, female gender, smoking, and treatment augmentation with magnesium. The parameters that increased the odds of remission were lower baseline HDRS scores, shorter history of disease, the presence of antidepressant-induced changes in the pharmaco-EEG profile at 6 h after treatment, and the fact of receiving treatment augmented with magnesium ions. The limitation of this study is a small sample size.

## 1. Introduction

According to the recent data from the World Health Organization, depression affects over 300 million people worldwide. By 2030, recurrent unipolar depressive disorders are projected to become the leading cause of the burden of disease worldwide, as calculated on the basis of Disability-Adjusted Life Years (DALYs) [1].

Despite having been studied for years, the etiology of depression is yet to be fully understood. More and more animal and clinical studies have suggested a role of the N-methyl-D-aspartate (NMDA) receptor complex and NMDA-mediated excitatory amino acid neurotransmission both in the pathophysiology and treatment of depression [2,3,4,5]. This concept seems to be confirmed by evidence of glutamate system abnormalities detected in the blood [6], cerebrospinal fluid [7], and brain tissue [8] of patients with depressive disorders. Modifying glutamatergic transmission by means of NMDA receptors is currently a promising target of antidepressant treatment [9].

The NMDA receptor complex is modified by multiple ligand binding sites. Recent years saw a number of experimental studies that confirmed an antidepressant effect of various NMDA receptor antagonists, such as ketamine, memantine, dextromethorphan, or MK-0657 [9]. However, the risk of severe side effects limits the use of these agents as antidepressant drugs [10].

One of the natural, inorganic modulators of the NMDA receptor complex are magnesium ions. They inhibit voltage-gated NMDA receptor channels at the same time inhibiting the flow of calcium ions. Moreover, they increase the expression of the GluN2B subunit of the NMDA receptor complex [11]. In the hippocampus, low magnesium levels in combination with high calcium and glutamate levels are believed to potentially cause functional changes in synapses, leading to the development of mood disorders, including depression [11,12]. There are several mechanisms responsible for antidepressant effects of magnesium. Apart from their direct NMDA-receptor antagonism, magnesium ions interact with other factors crucial in depression pathophysiology. Magnesium ions suppress hippocampal kindling and modulate protein kinase C [13]. Moreover, they affect P-glycoprotein (a protein responsible for blood-brain barrier permeability to glucocorticoids and other molecules), which alters the hypothalamic-pituitary-adrenal axis and damages the hippocampus [14]. Magnesium also plays a role in serotoninergic, noradrenergic, and dopaminergic neurotransmission [15] and it has an anti-inflammatory effect [16], which additionally increases its antidepressant potential.

Antidepressant properties of magnesium have been demonstrated in animal preclinical screen tests and models. Magnesium salts are active in the forced swim test (FST) as well as in olfactory bulbectomy and chronic mild stress models [17,18,19,20]. Furthermore, this bio-metal enhances antidepressant activity of standard antidepressants in the FST [15,21,22]. On the other hand, magnesium deficiency (induced by low-magnesium diet in laboratory animals) is related to depression-like behavior [23].

Multiple studies have demonstrated a relationship between depressive disorders and magnesium intake [24,25,26,27]. However, the data on the changes in magnesium levels in patients with depression are inconclusive. Some authors showed a positive correlation between magnesium levels and depression [28], whereas others showed a negative correlation [29]. Similar discrepancies were observed in the case of the severity of depression symptoms [30]. Nonetheless, Camardese et al. concluded that serum magnesium levels correlate with the response to treatment [31].

We would like to emphasize that the main goal in the treatment of a depressive episode is first to achieve a full therapeutic response and remission, followed by recurrence prevention, and ensuring the patient’s return to normal functioning [32]. One of the major problems in treating depression is the effectiveness of therapy. Some patients fail to achieve a satisfactory response to treatment. Initial antidepressant treatment, with adequate dosing and treatment duration, leads to remission only in 50% of patients [33]. Moreover, 20–30% of patients achieve incomplete remission, with some depressive symptoms persisting for a long time. Another therapeutic problem is a delay in therapeutic effects. All currently approved monoaminergic antidepressants exhibit latency in the therapeutic response, which considerably increases the risk of suicide and self-harm. Thus, there are unceasing attempts to potentiate and speed up the therapeutic effect [32].

Due to the limited effectiveness of antidepressant treatment, there is a great need for developing novel, satisfactory therapies. To date, there have been few clinical studies on magnesium supplementation in depressive disorders, and their findings have been inconclusive [34,35,36]. Therefore, the purpose of our study was to assess the efficacy and safety of antidepressant treatment accompanied with magnesium supplements.

## 2. Materials and Methods

Our 8-week study included 37 patients (admitted either to the Department of Psychiatry, Medical University of Warsaw or to Nowowiejski Hospital in Warsaw) who met the inclusion criterion of an ICD-10-codable depressive episode or major depression as defined in DSM-IV. The exclusion criteria were delusional disorders, organic disorders, high risk of suicide requiring electroconvulsive therapy, absolute contraindications for selective serotonin re-uptake inhibitors (SSRIs), absolute contraindications for magnesium ions, alcohol and substance abuse, and baseline pharmaco-EEG abnormalities. Patients with severe depression (more than 19 points in the HDRS) were included in the study. Patients were recruited without age or gender restrictions—adults over 18 years old.

All patients received fluoxetine at a daily dose of 20–40 mg. This standard treatment was augmented, in a double-blind manner, with either placebo or magnesium. The magnesium supplements used in this study were 40-mg magnesium effervescent tablets or powder containing 40 mg of magnesium (equivalent to 3.30 mEq of magnesium aspartate) administered 3 times per day.

The study was conducted in accordance with the Declaration of Helsinki. The protocol was approved by the Institutional Review Board and Bioethics Committee at the Medical University of Warsaw (KB/96/2006; KB/227/2012). All participants gave informed written consent prior to participating in this study.

Study participants were recruited based on baseline assessments, which included a physical examination (conducted by the same psychiatrist as those conducted later, throughout the study), psychometric scale score, and pharmaco-electroencephalography. Individuals who qualified to take part in the study underwent a one-week wash-out period (except in cases of previous SSRI treatment, where wash-out was extended to 6 weeks).

All study group patients were examined by the same physician at pre-defined time points: prior to treatment initiation (time 0), 6 h after the first dose of the drug (maximum serum concentration of the drug), and subsequently at 24 h, 2 weeks, 4 weeks, 6 weeks, and 8 weeks after treatment initiation [37].

The psychometric scales used in this study were the 21-item Hamilton Depression Rating Scale (HDRS), Hamilton Anxiety Rating Scale (HARS), and Clinical Global Impression Scale (CGIS). Treatment was considered effective when there was a 50% reduction in the baseline HDRS score. The cut-off HDRS score that defined remission was 6 or less [38].

The presence and severity of side effects were assessed based on history-taking, changes in Side Effect Rating Scale (SERS) scores as compared to baseline, and laboratory assessments, which were conducted at the same time as psychometric assessments. Any drugs that could affect the levels of the antidepressant were avoided during the study. When necessary, zopiclone (7.5 mg) or zolpidem (10 mg) was allowed every other day.

A high-performance liquid chromatography (HPLC) system (Shimadzu Corporation, Analytical Instruments Division, Kyoto, Japan) was used in this study to measure serum fluoxetine (FLU) and norfluoxetine (NFLU) levels. The measurement method was based on the reports by El-Yazigi and Raines [39], Aymard [40], Meineke [41], and Komorowska [42].

The following therapeutic ranges were adopted [42,43]: fluoxetine 50–450 ng/mL, norfluoxetine 50–350 ng/mL, fluoxetine and norfluoxetine 50–550 ng/mL. Serum fluoxetine levels were measured at the Psychopharmacology Laboratory of the Department of Psychiatry, WUM. Serum magnesium levels were measured by ALAB Laboratories and analyzed with the use of Hulanicki’s method [44]. The established ALAB reference range for serum magnesium levels (1.7–2.5 mg/dL) was adopted for this study. This was a double-blind study—with the principal investigator blinded to the magnesium levels in individual participants before study completion, as the laboratory reported only abnormalities in magnesium levels.

Pharmaco-EEG examinations were conducted prior to, and 6 and 24 h after, treatment initiation, and then at 2, 4, 6, and 8 weeks of treatment. The electroencephalograph used in this study was DigiTrack, version DTW (Elmico). Subsequently, EEG relative power spectra were calculated with NeuroGuide software using the fast Fourier transformation (FFT) algorithm. Adopting a 0.5-Hz resolution, we calculated the power spectra in delta (1.5–5.0 Hz), theta (5.5–8.0 Hz), alpha 1 (8.5–10.0 Hz), alpha 2 (10.5–12.0 Hz), beta 1 (12.5–18.5 Hz), beta 2 (19.0–20.5 Hz), and beta 3 (21.0–29.5 Hz) frequency bands. Arranged chronologically, *t*-test values for the individual bands formed a profile of EEG power spectrum changes over the treatment period.

Each of the graphs was classified by an expert, based on the presence or absence of an antidepressant-induced pharmaco-EEG profile. The following pharmaco-EEG profile, typical for tricyclic antidepressants (TCAs), was considered positive in fluoxetine-treated patients: an increase in high frequency beta waves (beta 3) [37,45,46].

All EEG examinations were performed at the Clinical Electroencephalography and Neurophysiology Laboratory of the Department of Psychiatry, WUM.

In the statistical analysis of our results, the Wilcoxon Rank-Sum Test for independent samples was used for the comparison of groups. Moreover, we used descriptive statistics and multivariate logistic regression models (GLIMMIX procedure), which allowed us to assess the odds ratios for an ineffective treatment and lack of remission with respect to each of the evaluated factors. The level of statistical significance was set at *p* < 0.05. All calculations were conducted with SAS 14.1.

## 3. Results

Seventeen (11 women [65%] and 6 men [35%]) out of the 37 participants included in the study received fluoxetine and magnesium, whereas 20 (10 women [50%] and 10 men [50%]) received fluoxetine and placebo. The mean age in the magnesium group (*n* = 17, group I) was 48.1 ± 15.5 years; the median age was 50 years; the age range was from 23 to 71 years, with 5 participants (29%) 60 years old or older. Body weight in this group ranged from 50.0 to 110.0 kg, with the mean of 71.2 ± 15.0 kg and median 70.0 kg. The mean height was 169.9 ± 10.0 cm (with the median of 168.0 cm and range of 158.0–192.0 cm). Mean disease duration at baseline was 5.6 ± 5.8 years (with the median of 4.0 years and range of 0.3–20.0 years). The mean age in the placebo group (*n* = 20, group II) was 49.7 ± 12.3 years; the median age was 52 years; the age range was from 24 to 65 years, with 6 participants (30%) 60 years old or older. The mean body weight in this group was 76.2 ± 16.1 kg (median 75.0 kg, range 48.0–112.0 kg). The mean height in this group was 171.9 ± 7.7 cm (median 173.5 cm, range 156.0–187.0 cm). Mean disease duration at baseline was 3.8 ± 4.5 years (median 2.0 years, range 0.4–16.0 years).

In group I, 10 participants (60%) were hospitalized once, 4 participants (23.5%) were hospitalized twice, one participant (5.9%) was hospitalized 3 times, one (5.9%) 4 times, and one participant (5.9%) was hospitalized more than 5 times. In group II, 15 participants (75.0%) were hospitalized once, 4 participants (20.0%) were hospitalized 2 times, one participant (5.0%) was hospitalized 5 times, and there were no participants hospitalized more than 5 times.

There were 2 non-smokers (11.8%) and 15 smokers (88.2%) in group I. The mean BMI in this group was 24.6 kg/m^2^ (median 24.2 kg/m^2^, range 19.1–35.5 kg/m^2^). There were 5 non-smokers (25.0%) and 15 smokers (75.0%) in group II. The mean BMI in this group was 25.8 kg/m^2^ (median 24.8 kg/m^2^, range 17.6–37.4 kg/m^2^).

Prior to treatment initiation, the mean HDRS score in group I (fluoxetine and magnesium) was 30.5 ± 6.0 (median 29; range 21–44) (Table 1). Other scales used in this study yielded the following scores prior to treatment initiation: HARS (mean score 20.1 ± 4.8, median 19, range 13–28), CGI (mean score 2.9 ± 0.7, median 3, range 2–4), and SERS (mean score 10.5 ± 3.4, median 10, range 6–18).

Prior to treatment initiation, the mean HDRS score in group II (fluoxetine and placebo) was 27.5 ± 5.5 (median 28; range 18–38) (Table 1). Other scales yielded the following scores at baseline: HARS scores: mean 18.5 ± 3.8, median 19, range 8–25; CGI scores: mean 3.1 ± 0.7, median 3, range 2–5; SERS scores: mean 11.3 ± 3.5, median 11, range 5–20.

There were no differences between groups at each examined time points in either HDRS (Table 1) or in CGI, HARS, and SERS scores (data not shown).

After 8 weeks of treatment, there was a 50% improvement in HDRS scores in 15 participants (88%) from group I and in 11 participants (73%) from group II. There was no significant difference between the groups in terms of treatment efficacy.

Remission, which had been pre-defined as HDRS score reduction to 6 points or less, was achieved in 6 participants (35%) from group I and in 4 participants (27%) from group II. There was no significant difference between the groups in terms of remission rates.

**Figure 1:**
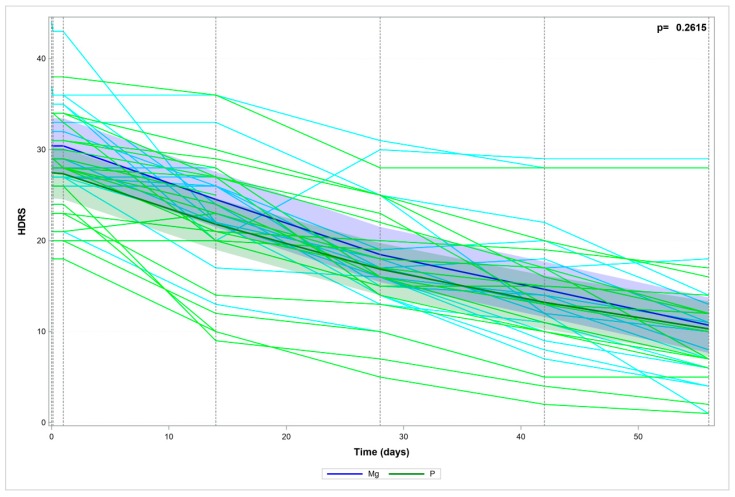
Hamilton Depression Rating Scale (HDRS) scores over time—the measured data and trend.

**Figure 2:**
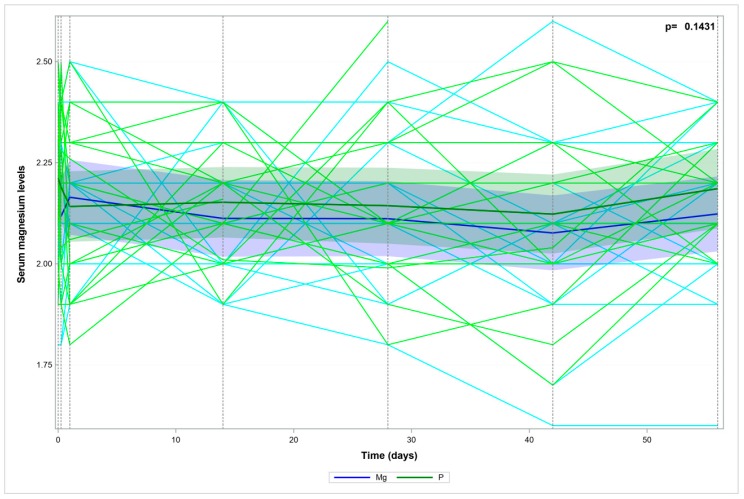
Serum magnesium levels over time—the measured data and trend.

The two study groups (I and II) showed no significant differences in terms of HDRS score changes during treatment (Figure 1). The two study groups (I and II) showed no significant differences in terms of serum magnesium levels during treatment (Figure 2).

Models of multivariate analysis were used to calculate odds ratios for both remission (model 1) and treatment efficacy (model 2) using the compared values of each evaluated parameter.

Model 1 (Table 2), which was used to analyze the odds of remission, included the following parameters: the baseline HDRS score; disease duration; pharmaco-EEG profile (obtained 6 h after treatment initiation) showing evidence of TCA use (yes vs. no); and the type of treatment (magnesium vs. placebo).

**Figure 3:**
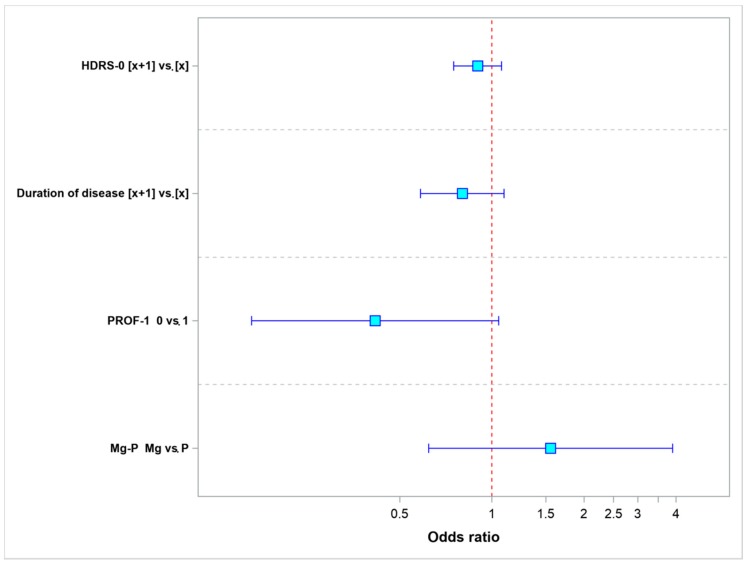
Odds ratio values for remission.

Figure 3 shows the odds ratios (ORs) for remission (with 95% confidence intervals) for the individual parameters. A statistically significant parameter would have its OR = 1.0 value positioned completely beyond the confidence interval. An odds ratio equal to 1.0 means that both compared values of the given parameter yield identical odds of remission.

We found that an increase in the HDRS score by 1 point as compared to baseline, disease duration longer by 1 year, and a lack of antidepressant-induced changes in the pharmaco-EEG profile at 6 h after treatment decrease the odds of remission. The use of magnesium to augment the effect of treatment increases the odds of remission.

**Figure 4:**
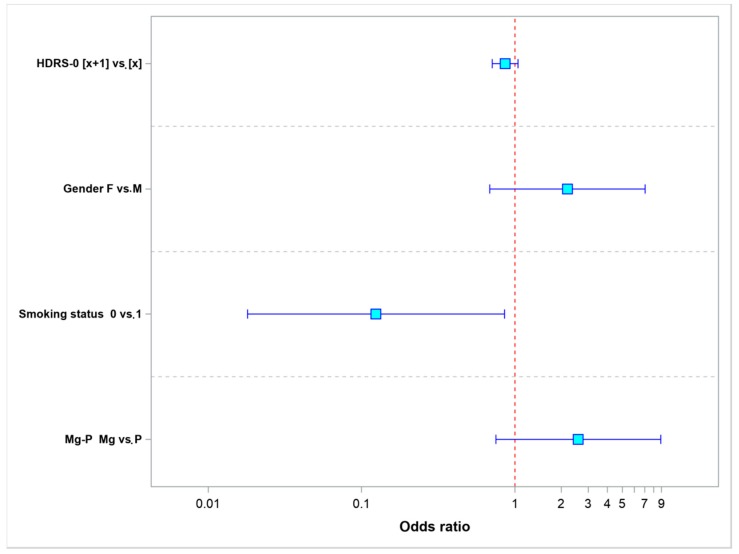
Odds ratio values for treatment efficacy.

Model 2 (Table 3)—which was used to analyze treatment efficacy, included the following parameters: the baseline HDRS score; the patients’ gender; smoking status; and the type of treatment (magnesium vs. placebo). Figure 4 shows the increase in the baseline HDRS score by 1 point and being a non-smoker (*p* = 0.0034) reduced the odds of effective treatment. The female sex and the use of magnesium to potentialize the effect of fluoxetine increased the odds of effective treatment.

## 4. Discussion

Our study evaluated the efficacy and safety of antidepressant treatment augmented with magnesium. Despite the fact that the fluoxetine-and-magnesium group showed higher rates of 50% improvement in HDRS scores at week 8 than the fluoxetine-and-placebo group, the difference was not statistically significant. Similar, though also non-significant, results were obtained while evaluating remission. Moreover, the magnesium and placebo groups showed no significant differences in terms of the efficacy and safety of treatment at any evaluated time point.

Our findings are unlike those reported in some earlier studies. Tarleton et al. demonstrated a decrease in depression symptoms already after a two-week period of magnesium supplementation at 248 mg per day. Their study employed the PHQ-9 for the diagnosis of depression [47], with 126 participants with mild-to-moderate depression symptoms included in the study. Their results showed no relationship with the participants’ age, gender, baseline disease severity, or antidepressant treatment [48]. Earlier reports by Tarleton et al. also showed a relationship between low magnesium intake (<184 mg/day) and depression [26]. This observation was also supported by a Finnish study including exclusively males (2320 participants). That study demonstrated that adequate magnesium intake may prevent depression [27]. In light of the available studies, the reports of major depression cases described by Eby et al. seem very interesting, as they showed a rapid (within under 7 days) recovery in response to treatment with magnesium (in the form of glycinate and taurinate) at 125–300-mg administered with every meal and before sleep [35]. Moreover, another two-week randomized study in a group of 23 elderly patients with newly diagnosed depression, associated with type 2 diabetes and hypomagnesemia, showed treatment with magnesium chloride (450 mg/day) to be equally effective as that with imipramine (50 mg/day) [49].

Nonetheless, our findings were consistent with those of some other authors. Mehdi et al. observed no changes in HDRS scores following magnesium supplementation in a group of participants (*n* = 12) with mild-to-moderate treatment-resistant depression [50]. Similar results were observed in a randomized clinical study in a group of female patients with postpartum depression, where an 8-week treatment with magnesium at 320 mg/day failed to reduce the symptoms of depression evaluated with the Edinburgh Postnatal Depression Scale [51]. Moreover, a large Spanish study conducted in a Mediterranean population (15,836 participants) showed no conclusive evidence to support the claim that an increased magnesium intake might be associated with a lower risk of developing depression [52]. Due to these conflicting findings, the efficacy of magnesium in antidepressant treatment is still unknown.

Table 4 presents a compilation of clinical studies on the use of magnesium in the treatment of depressive disorders as well as data on the study methods used.

We would like to emphasize the fact that the tools for assessing depression symptoms which were used in the studies cited above were non-uniform, with the following being the most commonly used questionnaires: the Human Population Laboratory (HPL) Depression Scale [27], Patient Health Questionnaire (PHQ-9), Yasavage and Brink Scale [49], and Hamilton Depression Rating Scale (HDRS) [50]. It seems obvious that these psychometric instruments differ in terms of accuracy, reliability, or standardization. Therefore, the selection of an appropriate questionnaire may determine the study findings. Consistent with our observations, a study by Mehdi et al. showed no significant changes in HDRS scores following magnesium supplementation; however, increased serum magnesium levels correlated with a clinical improvement measured with the PHQ-9 [50]. The questionnaire used in our study is currently considered to be the gold standard among psychometric tools used to assess the severity of depression. Additionally, it helps precisely monitor the patient’s condition during antidepressant treatment. The HDRS is an observer-rated instrument, which means that the assessment is conducted by an experienced physician who knows the standards in symptom severity assessment. Unlike self-administered questionnaires used by other investigators, the HDRS is considered to be a more objective and accurate tool. The methods of assessing a response to treatment in our study additionally included looking for antidepressant-induced changes in pharmaco-EEG profiles at various time points. Our results showed no statistically significant differences between the study groups. The discrepancies between our findings and the ones reported by other authors may be also due to a varied duration of magnesium supplementation and different assessment time points.

Other factors that may affect treatment efficacy include both the dosage and form of magnesium supplements. In all the studies mentioned above where oral magnesium supplementation was shown to decrease depression symptoms magnesium doses ranged from 125 to 900 mg/day. In all these cases the dosage was higher than that used in our study. Currently, there are a number of available magnesium formulations. The key criterion that makes these formulations different is their bioavailability. Due to the fact that only some (30–40%) of the ingested magnesium is absorbed by the body, it is very important to conduct magnesium supplementation using formulations characterized by better absorption. Magnesium aspartate, citrate, lactate, and chloride are considered to have a higher bioavailability in comparison with that of either magnesium oxide or sulfate [11]. In the present study dose of magnesium (120 mg Mg^2+^/day) was chosen as the acceptable (and widely applied) in Poland (indicated by Pharmindex—polish drug encyclopedia). Despite the fact that we used magnesium aspartate characterized by high bioavailability and a structure similar to that of magnesium compounds found in a normal diet, we observed no significant improvement in depression symptoms. Moreover, since our study was conducted exclusively in a Polish population, we cannot exclude the potential effects of genetic and environmental factors.

Despite the well-documented relationship between magnesium and depression, its mechanisms are still unknown. Thus, the role of magnesium in antidepressant treatment augmentation is difficult to elucidate. A study by Camardese et al. demonstrated a more pronounced response to antidepressant treatment in patients with higher magnesium levels [31]. Another study showed a marked increase in intracellular magnesium levels following treatment with either amitriptyline or sertraline. It is precisely this increase in intracellular magnesium levels that has been suggested to be partially responsible for the effect of antidepressant drugs [53].

Experimental pre-clinical studies on animal models demonstrated that the antidepressant effect of magnesium is a result of its role in serotoninergic neurotransmission [54]. The potential synergism between magnesium and antidepressants warrants further studies on antidepressant treatment potentialization.

Further studies are necessary to discover whether magnesium supplementation can justify the use of antidepressants at lower doses or help avoid the necessity of combination regimens.

We would like to emphasize our multivariate analysis results that identified the parameters that increased the odds of remission and treatment efficacy. The parameters increasing the odds of remission (i.e., HDRS score of <6) included lower baseline HDRS scores, shorter history of disease, the presence of antidepressant-induced pharmaco-EEG profile at 6 h after drug administration, and treatment augmentation with magnesium ions. The parameters increasing the odds of treatment efficacy were lower baseline HDRS scores, female gender, being a smoker, and treatment augmentation with magnesium ions. It seems obvious that an earlier diagnosis and lower symptom severity are favorable prognostic factors. The benefits of magnesium supplementation have been widely discussed. One of the arguments explaining the better treatment efficacy in females observed in our study may be other authors’ reports of generally lower magnesium levels in females [55,56]. Pregnancy and chronic use of oral contraceptives are known to lead to hypomagnesemia [57]. Moreover, magnesium supplementation has been shown to prevent the development of postpartum depression [35].

We would like to emphasize that one of the parameters shown to significantly increase the odds of effective treatment was the fact of being a smoker. Nicotine is known to directly affect mood in humans. The relationship between depression and smoking has been extensively studied, and the findings demonstrated antidepressant properties of nicotine [58,59,60]. Smokers with a history of depression who refrain from smoking have a higher risk of developing another depressive episode [61]. Salin-Pascual et al. observed an improvement in the mood of non-smoking participants with major depression following the use of nicotine patches [62]. This effect is most likely associated with dopaminergic reward system activation [63] and serotoninergic neurotransmission [60]. The results of our study demonstrate potentially antidepressant effects of nicotine. However, our findings need to be confirmed in a larger number of patients.

Despite the fact that our study did not conclusively demonstrate an increased efficacy of antidepressant treatment augmented with magnesium, magnesium supplementation helped predict treatment efficacy and remission. Further studies are necessary to assess whether magnesium supplementation may be a valuable complement to standard antidepressant treatments.

## 5. Conclusions

The magnesium and placebo groups showed no statistically significant differences in terms of HDRS scores, serum magnesium levels, treatment safety and efficacy, or pharmaco-EEG profiles. Nonetheless, supplementation with magnesium ions is one of the parameters that help increase the chances of treatment efficacy and remission. The limitation of this study is the small sample size.

## Figures and Tables

**Table 1:** Effect of magnesium or placebo supplementation on Hamilton Depression Rating Scale (HDRS) scores in patients treated with fluoxetine.

Group I: Magnesium							Group II: Placebo						
Variable	N	Mean	SD	Med	Min	Max	N	Mean	SD	Med	Min	Max	*p*-Value
HDRS_0	17	30.5	6	29	21	44	20	27.5	5.5	28	18	38	0.1120
HDRS_6H	17	30.4	5.8	29	21	43	20	27.5	5.5	28	18	38	0.1197
HDRS_24H	17	30.4	5.8	29	21	43	20	27.4	5.4	28	18	38	0.1059
HDRS_2W	17	24.5	5.6	24	13	36	20	21.8	7.5	23	9	36	0.2237
HDRS_4W	17	18.5	5.9	17	10	31	17	17.2	6.4	17	5	28	0.5605
HDRS_6W	17	14.6	6.9	13	5	29	16	13.4	6.6	14	2	28	0.6100
HDRS_8W	17	10.7	7.9	8	1	29	15	10.4	6.8	10	1	28	0.9080

N—number of patients; SD—standard deviation; Med—Median; Min—minimum; Max—maximum HDRS: Hamilton Depression Rating Scale; HDRS_0—scores before treatment; H—hours; W—week. The Wilcoxon Rank-Sum Test for independent samples was used for the comparison of groups.

**Table 2:** Odds ratio values for the parameters evaluated in this model—the odds ratio for remission.

Parameter	Values	Odds Ratio	95% LCL	95% UCL	*p*-Value
HDRS-0	[*x* + 1] vs. [*x*]	0.8982	0.7503	1.0752	0.2422
Duration of disease	[*x* + 1] vs. [*x*]	0.8001	0.5842	1.0956	0.1643
PROF-1	0 vs. 1	0.4151	0.1637	1.0527	0.0641
Mg-P	Mg vs. P	1.5545	0.6206	3.8938	0.3464

LCL lower confidence limit; UCL upper confidence limit.

**Table 3:** Odds ratio values for the parameters evaluated in this model—the odds of achieving 50% improvement in HDRS scores (efficacy).

Parameter	Values	Odds Ratio	95% LCL	95% UCL	*p*-Value
HDRS-0	[*x* + 1] vs. [*x*]	0.8614	0.7093	1.0461	0.1321
Gender	F vs. M	2.1942	0.6840	7.0392	0.1864
Smoking status	0 vs. 1	0.1242	0.0181	0.8530	0.0339
Mg-P	Mg vs. P	2.5869	0.7510	8.9111	0.1320

LCL, lower confidence limit; UCL, upper confidence limit.

**Table 4:** Effect of magnesium (Mg) supplementation in human depression.

Depression Type	Type of Study	N	Dose of Mg^+2^ mg/day p.o.	Salt	Effect	References
Major depression	Case	4	125–300	Glycinate taurinate	+	[28]
Depression (early, type 2 diabetes, hypomagnesemia)	Randomized Mg vs. IMI|	23	450	Chloride	+	[42]
Depression Gitelman’s syndrome, (hypomagnesemia)	Case	1	300–900 plus intravenous 1200	Oxide sulfate	+	[46]
Major depression (hypomagnesemia)	Placebo	60	300	Oxide	+	[47]
Major depression	Placebo cross-over	112	248	Chloride	+	[41]
Postpartum depression	Placebo	66	87	Sulfate	−	[44]
Major depression	Placebo	37	120	Aspartate	−	present study
Major depression (TRD)	Monotherapy	12	Intravenous 1000	Sulfate	−	[43]

N—number of patients; + positive effect of Mg supplementation; − no effect of Mg supplementation.
